# Conservation of Archaeal C/D Box sRNA-Guided RNA Modifications

**DOI:** 10.3389/fmicb.2021.654029

**Published:** 2021-03-12

**Authors:** Ruth Breuer, Jose-Vicente Gomes-Filho, Lennart Randau

**Affiliations:** Prokaryotic RNA Biology, Philipps-Universität Marburg, Marburg, Germany

**Keywords:** C/D box, methylation, RNA modification, RNA folding, RNA structure

## Abstract

Post-transcriptional modifications fulfill many important roles during ribosomal RNA maturation in all three domains of life. Ribose 2'-*O*-methylations constitute the most abundant chemical rRNA modification and are, for example, involved in RNA folding and stabilization. In archaea, these modification sites are determined by variable sets of C/D box sRNAs that guide the activity of the rRNA 2'-*O*-methyltransferase fibrillarin. Each C/D box sRNA contains two guide sequences that can act in coordination to bridge rRNA sequences. Here, we will review the landscape of archaeal C/D box sRNA genes and their target sites. One focus is placed on the apparent accelerated evolution of guide sequences and the varied pairing of the two individual guides, which results in different rRNA modification patterns and RNA chaperone activities.

## Introduction

The chemical modification of RNA has long been known to play a role in a wide variety of cellular processes in all three domains of life. The manifold modifications can be introduced co- or post-transcriptionally and concern all classes of RNA molecules. The most abundant RNA modification is the ribose-2'-*O*-methylation, which is commonly found on ribosomal RNAs (rRNAs) and transfer RNAs (tRNAs) and also present on small nuclear RNAs (snRNAs) in archaea and eukaryotes (Maden et al., 1995; Kiss-László et al., 1996; Tycowski et al., 1998; Omer et al., 2000; Vitali and Kiss, 2019). This modification fulfills many different functions: It can protect RNA from ribonucleolytic cleavage, stabilize single base pairs, exhibit a chaperone function and influence folding at high temperatures (Kawai et al., 1992; Herschlag et al., 1993; Williams et al., 2001; Helm, 2006). The latter function is especially important in thermophilic organisms, therefore, it is no surprise that thermophilic archaea exhibit a significantly larger number of 2'-*O*-methylations than mesophilic archaea (Noon et al., 1998; Omer et al., 2000; Su et al., 2013).

**Figure 1:**
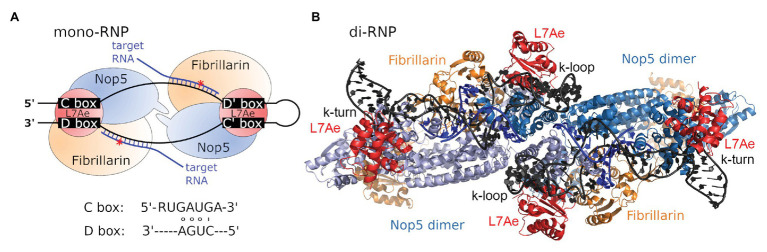
C/D box sRNP architecture. **(A)** Schematic view of the archaeal C/D box mono-sRNP with bound target RNA (blue), consisting of the C/D box sRNA (black), L7Ae (red), Nop5 (blue), and fibrillarin (orange). The red asterisk denotes the position of methylation. Consensus C and D box sequences are indicated. **(B)** The C/D box sRNP complex has been observed to exist as a dimeric variant consisting of two C/D box sRNAs and four copies of each protein (pdb-id:4BY9; Lapinaite et al., 2013).

In bacteria, 2'-*O*-methylations are comparatively rare and introduced by site- or region-specific protein-only enzymes (Decatur and Fournier, 2002). In contrast, methylation of the ribose moiety is more commonly observed in archaea and eukaryotes, which both utilize an RNA-dependent mechanism involving so-called C/D box s(no)RNAs. Here, the methylation reaction is performed by a ribonucleoprotein (RNP) complex carrying a small nucleolar RNA (snoRNA), or its archaeal homolog, the sno-like RNA (sRNA; Figure 1A; Lischwe et al., 1985; Ochs et al., 1985; Filipowicz and Kiss, 1993; Maxwell and Fournier, 1995; Gaspin et al., 2000; Omer et al., 2000).

C/D box sRNAs were found to be approximately 50–70 nt long in archaea and between 50 and 300 nt long in eukaryotes (Lui and Lowe, 2013). These RNA molecules are named for four conserved sequence elements: the box C and box C' motifs with the consensus sequence RUGAUGA and the box D and box D' motifs with the consensus sequence CUGA (Maxwell and Fournier, 1995; Kiss-Laszlo et al., 1998). During C/D box sRNA folding, the motifs C and D base-pair and form a helix-loop-helix structure termed kink-turn (k-turn). The k-turn is a short stem structure comprising non-canonical base pairs and carrying two sheared base pairs (AG and GA) at its top (Watkins et al., 2000; Klein et al., 2001). Base pairing between the motifs C' and D' results in a similar structure called the k-loop, which consists of a single stem closed by a terminal loop (Nolivos et al., 2005). The sequences located between the motifs C and D', and C' and D, are complementary to the target RNA sequences and therefore serve as guides for the identification of methylation sites (Figure 1A). The length of the archaeal guide sequences ranges from 10 to 12 nt. Target methylation occurs at the nucleotide complementary to the fifth nucleotide upstream of the box D/D' motif (Kiss-László et al., 1996; Tran et al., 2005). A recent study in *Drosophila* identifies the minimal functional eukaryotic C/D box snoRNA as a single-domain molecule with (i) a terminal stem with a consensus k-turn domain, (ii) one box C and one box D separated by a 14 nt long antisense element and (iii) a one-nucleotide spacer between box C and the antisense element (Deryusheva and Gall, 2019).

Interestingly, archaeal organisms harbor not only linear, but also circular C/D box sRNAs, though their role remains to be determined (Starostina et al., 2004; Danan et al., 2012; Randau, 2012; Su et al., 2013). The analysis of permuted RNA-seq reads allowed for the detection of circularization junctions of RNA molecules and revealed that C/D box sRNA termini can be fused. Inspection of these fusion sites indicated that the termini are not clearly defined, but can vary by few nucleotides for individual C/D box sRNA species. In addition, linear C/D box sRNAs are usually observed in parallel to circular variants. Notably, in *Sulfolobus solfataricus*, C/D box sRNAs occur predominantly in the linear form, whereas in *Pyrococcus furiosus* almost all C/D box sRNAs exist in both linear and circular forms with similar abundance (Starostina et al., 2004; Danan et al., 2012). Furthermore, archaeal circular RNA molecules exist among tRNA introns and rRNA processing intermediates (Danan et al., 2012; Jüttner et al., 2020). *Thermoproteus* species were found to require circularization of signal recognition particle (SRP) RNAs to yield functional molecules (Plagens et al., 2015). In these cases, the 5' and 3' ends of the RNA molecule fold into close contact and form a bulge-helix-bulge motif which is recognized and cleaved by the tRNA splicing endonuclease and subsequently ligated by the tRNA ligase RtcB (Trotta et al., 1997; Englert et al., 2011; Popow et al., 2011). However, C/D box sRNA termini usually do not form canonical BHB motifs and the exact method of circularization remains unclear (Starostina et al., 2004). Since circular sRNA molecules have been nearly exclusively found in thermophiles thus far, it is suggested that the circularization provides stability at elevated growth temperatures (Starostina et al., 2004; Danan et al., 2012). Here, it is plausible that the close proximity of C/D box sRNA termini upon protein binding facilitates RNA ligation, representing a statistic event that is positively selected for due to the increased stability of the circularized products.

The C/D box sRNA is part of the C/D box RNP complex which contains three highly conserved proteins in archaea and four proteins in eukaryotes (Figure 1A). Upon adopting its secondary structure, the k-turn and k-loop of the C/D box sRNA are bound and stabilized by the RNA-binding protein L7Ae (Snu13/15.5 K in yeast/human; Kuhn et al., 2002; Omer et al., 2002; Gagnon et al., 2010). Binding of the C/D box sRNA by L7Ae depends on three essential features: (i) the terminal stem at the 5' and 3' ends of the C/D box sRNA, which juxtaposes the boxes C and D motifs, (ii) two sheared GA base pairs formed by pairing of the box C and box D motifs, and (iii) the box C uridine which is part of the k-turn’s internal loop (Kuhn et al., 2002). After binding of the C/D box sRNA by L7Ae, the assembly of the RNP is completed by binding of the proteins Nop5 (Nop56/Nop58 heterodimer in yeast and humans) and fibrillarin (Nop1/fibrillarin in yeast/human; Omer et al., 2002; Bortolin et al., 2003). The N-terminal and C-terminal domains of Nop5 interact with fibrillarin and the C/D box sRNA, respectively. Furthermore, the coiled-coil domain of Nop5 mediates Nop5-dimerization for optimal interaction with the C/D box sRNA (Aittaleb et al., 2003).

Fibrillarin exhibits a conserved *S*-adenosyl-methionine (SAM) binding motif and possesses methyl transfer activity. It was found that this activity is dependent on C/D box sRNP formation and could not be observed independent of the complex (Wang et al., 2001; Omer et al., 2002). For the 2'-*O*-methylation reaction, fibrillarin uses *S*-adenosyl-L-methionine as a methyl group donor and after depositing the methyl group at the 2'-OH moiety, the ribose preferably adopts an endo-conformation, thereby blocking sugar-edge interactions (Kawai et al., 1992; Auffinger and Westhof, 1997; Hansen et al., 2002; Motorin and Helm, 2010). Conversely, a fibrillarin-Nop5 heterodimer of *Pyrococcus abyssi* was recently found to perform *in vitro* 2'-*O*-methylation of rRNA independently of L7Ae and C/D box sRNAs (Tomkuviene et al., 2017). In C/D box sRNPs containing fibrillarin, recent evidence shows that the guide RNA sequence determines the affinity of fibrillarin for the substrate and the extent of fibrillarin binding correlates with the efficiency of methylation (Graziadei et al., 2020).

First reports of the structure of the C/D box sRNP complex provided contradictory results for arrangement and number of associated proteins. However, it soon became clear that observed differences were caused by the type of C/D box sRNA that had been utilized in the *in vitro* experiments. While the usage of an artificial two-stranded RNA lacking the k-loop motif lead to the assembly of a monomeric complex consisting of one RNA and two copies of each protein, the usage of an *in vitro* transcribed natural C/D box sRNA sequence lead to the assembly of dimeric complex consisting of two RNAs and four copies of each protein (Figure 1B; Bleichert et al., 2009; Bleichert and Baserga, 2010; Xue et al., 2010; Lin et al., 2011; Bower-Phipps et al., 2012; Lapinaite et al., 2013). Accordingly, these results suggest that the nature of the RNA determines if mono- or diRNPs are assembled and influences these complexes’ functional roles. These and other findings on the structural diversity of C/D box sRNPs are extensively reviewed by Yu et al. (2018).

## C/D Box sRNA Targets

### Ribosomal Targets

A first study aiming to identify archaeal sRNAs employed co-immunoprecipitation with archaeal fibrillarin and Nop5 and identified 18 C/D box sRNAs in *Sulfolobus acidocaldarius*. Furthermore, methylations at the predicted target positions for six of these sRNAs were verified using deoxyribonucleotide triphosphate (dNTP) concentration-dependent primer extension assays (Omer et al., 2000). Subsequent experiments lead to the discovery of over 200 sRNAs across seven archaeal species, targeting mostly – though not exclusively – archaeal rRNAs. Here, it was also revealed that, in contrast to eukaryotes, most archaeal sRNAs possess two sequences able to guide methylation and that these double guides can target closely linked positions on the same RNA molecule (Omer et al., 2000). At the same time, another study reported the identification of a family of 46 archaeal sRNAs in the genomes of three species of the hyperthermophile *Pyrococcus* species Additionally, these sRNAs were experimentally verified in *P. abyssi* using Northern hybridization (Gaspin et al., 2000).

Shortly afterwards, another study used a combination of MALDI-MS and primer extension assays to locate conserved modification patterns in the A-loop region of the 23S rRNA in five archaeal and eubacterial species. The A-loop of the 23S rRNA (also known as helix 92), constitutes part of the peptidyl transferase loop in domain V of the 23S rRNA and its functional importance has been emphasized by several studies (Hansen et al., 2002). In fact, loss of the 2'-O-ribose methylation at position U2552 in the A-loop leads to decreased growth rate and reduced protein synthesis activity in *Escherichia coli* (Caldas et al., 2000). It was shown that despite variation in the exact positions of modifications in the helices 90–92, modifications in the A-loop are always present at positions equivalent to U2552 and/or G2553 in *E. coli* (Hansen et al., 2002). Projecting these previously identified modifications from *E. coli* onto their corresponding positions in the 2.4 Å X-ray crystal structure of the *Haloarcula marismortui* 50S ribosome subunit, all modifications were found to be clustered around the peptidyl transferase center (Ban et al., 2000; Hansen et al., 2002).

**Figure 2:**
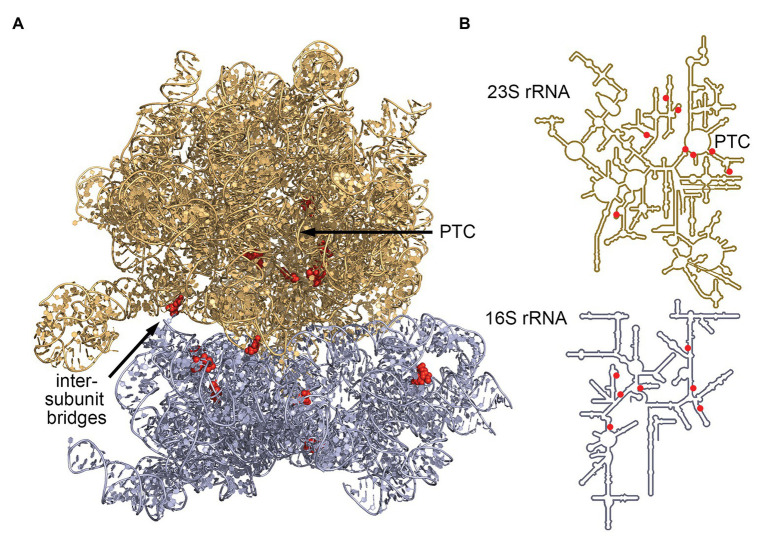
Most conserved C/D box sRNA guide targets of archaeal 16S and 23S rRNA. Analysis of C/D box sRNA guides of seven archaeal species (Dennis et al., 2015) identified seven 16S rRNA sites and eight 23S rRNA sites that are targeted by a minimum of four guides. These sites (red) are clustered at the ribosome core [peptidyl-transferase center (PTC)] and at the intersubunit bridges. **(A)** Positions were mapped onto the ribosome structure of *Thermococcus kodakarensis* (pdb-id: 6SKF; Sas-Chen et al., 2020) and **(B)** onto the secondary structure representations of archaeal rRNAs (Petrov et al., 2014).

The advent of RNA-seq has enabled researchers to efficiently identify C/D box sRNAs among any organism’s total RNA pool. Subsequently, their guide sequences can be used to computationally predict their potential RNA targets on the basis of their hybridization potential. Analyses of RNA-seq coverage revealed large numbers of abundantly transcribed small RNAs with readily identifiable C and D box sequences. These postulated C/D box sRNAs were, for example, described for model archaea of different archaeal phyla: *Nanoarchaeum equitans* (26 C/D box sRNAs), *Ignicoccus hospitalis* (128 C/D box sRNAs), *Methanococcus maripaludis* (7 C/D box sRNAs), *Methanopyrus kandleri* (127 C/D box sRNAs), *Pyrobaculum calidifontis* (88 C/D box sRNAs), *S. acidocaldarius* (61 C/D box sRNAs) and *Thermoproteus tenax* (52 C/D box sRNAs). Using the guide sequences of these C/D box sRNAs, 719 potential 2'-*O*-methylation sites in the archaeal 23S and 16S rRNA sequences were identified and hinted at common targets and rRNA regions (Figure 2). This dataset revealed some shared methylation targets but did not reveal a single position to be uniformly present in all seven species. Instead, it became clear that methylation targets cluster in hotspot regions of the rRNA molecules. Among all investigated species, these methylation hotspots have been detected in the functionally important and evolutionary conserved regions of the ribosome (Liang et al., 2009; Dennis et al., 2015; Lui et al., 2018).

The archaeal consensus 23S rRNA structure exhibits six domains surrounding a central core. Conserved methylation hotspots are identified in domain II helices 35 and 35a, domain IV helices 61 and 68–71, and domain V helices 90–93 (Dennis et al., 2015). These regions correspond to the ancient core of the ribosome where domain V lies at the center of the large ribosomal subunit. One major cluster of hotspots surrounds the catalytic peptidyl transferase center located in domain V, where peptide bond formation and peptide release occurs (Figure 2; Petrov et al., 2013; Dennis et al., 2015; Lui et al., 2018). Another predicted cluster lies in domain IV where helices 68, 69, and 71 form part of the interface between the large and the small ribosomal subunits (Cate et al., 1999; Dennis et al., 2015; Lui et al., 2018).

The archaeal consensus 16S rRNA structure consists of four domains connected by a central core which is located close to the functional decoding center of the small ribosomal subunit (Wimberly et al., 2000). Here, conserved methylation hotspots are predicted in helices 3, 18, and 27 (Dennis et al., 2015). In fact, helix 18 is the core of the decoding center and responsible for monitoring the codon-anticodon pairings (Ogle et al., 2001). Due to their location, the methylated nucleotides likely contribute to stabilizing the decoding center as well as the association of the four domains. Furthermore, predicted methylation clusters are especially dense in regions which are not protected by RNA-binding proteins. It has therefore been proposed that the modifications help stabilize the structure of these exposed regions and in turn support subunit interactions (Dennis et al., 2015). These findings are corroborated by a study across six *Pyrobaculum* species, revealing that most rRNA-targeting C/D box sRNAs are dual guides targeting sites within 100 nt of each other (Lui et al., 2018).

### Non-ribosomal Targets

The initial prediction and experimental verification of archaeal rRNA targets of C/D box sRNAs revealed additional antisense elements matching tRNAs (Gaspin et al., 2000; Omer et al., 2000). Further investigation revealed the presence of four C/D box sRNAs targeting the first position of the anticodon of the tRNAs tRNA-Leu (CAA), tRNA-Leu (UAA), tRNA-Met and tRNA-Trp in three *Pyrococcus* species. One of these sRNAs, termed sR50, corresponds to the intron of its predicted target, the pre-tRNA-Trp and was shown to guide the methylation of the pre-tRNA-Trp nucleotides Cm34 and Um39 in *in vitro* experiments in *Haloferax volcanii* (Omer et al., 2000; D’Orval et al., 2001). This proposed *cis*-acting mechanism was later shown to be a *trans*-acting or intramolecular mechanism by a study which also revealed the sequential pattern of C/D box sRNA-guided methylation (Bortolin et al., 2003; Singh et al., 2004). Recently, an in-depth analysis of C/D box sRNA families in *Pyrobaculum* species computationally predicted tRNA targets for 16% of the identified guide sequences (as opposed to 56% rRNA targets; Lui et al., 2018). Unsurprisingly, the tRNA methylation targets correspond to structurally conserved regions, however, in contrast to rRNA methylation *via* double-guide sRNAs, tRNA methylation is mediated by sRNAs where one guide targets the tRNA while the other guide has no target. Interestingly, a tRNA-targeting sRNA can mediate methylation of either a single or many tRNAs, depending on whether its guide targets a unique sequence like the region surrounding the wobble base in the anticodon (position 34) or a conserved sequence shared across different tRNA families (Lui et al., 2018). Conversely, a few tRNAs in *S. acidocaldarius* and *P. furiosus* contain several methylated or predicted methylated nucleotides which have not yet been linked to corresponding sRNAs or stand-alone specific methyltransferases (Wolff et al., 2020).

Several computational studies also identified numerous “orphan guides,” which are defined as snoRNA guide sequences that do not show complementary to any known RNA target (Hüttenhofer et al., 2001; Yang et al., 2006; Lui et al., 2018). Looking for target RNA sequences outside of rRNAs or tRNAs has relieved many guide sequences of their “orphan status,” thereby contributing to the expanding functional diversity of snoRNAs (see also Discussion section and reviewed in Falaleeva et al., 2017; Bergeron et al., 2020). At least some orphan guides can be considered as a consequence of accumulated mutations in the guide sequence, eventually leading to the evolution of guides for novel methylation targets (Dennis et al., 2001; Lui et al., 2018).

### C/D Box sRNAs and Their Role as RNA Chaperones

Shortly after their discovery, it was suggested that C/D box sRNAs might function as RNA chaperones (Steitz and Tycowski, 1995). This theory gained further support by a subsequent computer simulation of long-range rRNA interactions at elevated temperatures in the presence of double-guided C/D box sRNAs (Schoemaker and Gultyaev, 2006). Indeed, there exist several C/D box sRNAs whose guide sequences target positions at a considerable distance from each other. For example, *N. equitans* exhibits two instances where the predicted targets are distant in sequence but close in the secondary structure: the guides of sR17 target nucleotides situated on opposing strands of helix 28, the defining helix of domain III of the 16S rRNA and the guides of sR15 target nucleotides on opposing strands of helix 30 which defines a large subsection of domain III (Dennis et al., 2015). These findings lead to the conclusion that the C/D box sRNAs act as chaperones by bringing distant rRNA sequences together and facilitate their annealing, thereby assisting in ribosome subunit assembly (Gaspin et al., 2000; Dennis et al., 2015).

## C/D Box sRNA Gene and Guide Evolution

### Genomic Context Variability

In yeast, most snoRNA genes are transcribed from independent RNA polymerase II or III promoters as mono- or polycistronic transcripts (Li et al., 2005; Dieci et al., 2009). In plants, the C/D box sRNA genes exist almost exclusively as polycistronic clusters, or, to a lesser extent, as dicistronic tRNA-C/D box snoRNAs (Leader et al., 1997; Kruszka et al., 2003; Barbezier et al., 2009). In vertebrates, independent promoters are rare and snoRNA genes are usually located in introns of protein-coding or non-protein-coding genes (Pelczar and Filipowicz, 1998; Weber, 2006) and only few of them exist as polycistrons (Leader et al., 1994; Tycowski et al., 2004). The polycistronic transcripts or intron-located C/D box snoRNAs are processed and matured by endo- and exoribonucleolytic activities (Caffarelli et al., 1994; Kiss and Filipowicz, 1995; Villa et al., 1998).

In archaea, analysis of the genomic context of C/D box sRNA genes revealed a variable organization of promoter and processing elements. A 2017 study on the genomic context of C/D box sRNAs in six archaeal model organisms concluded that only a minority of archaeal C/D box sRNAs are transcribed from independent promoters as only 20% of all investigated genes exhibited a conserved, TATA box-like motif in their 50 nt upstream region (Tripp et al., 2017). Instead, the majority of C/D box sRNA genes overlap with either the 5' or the 3' end of a neighboring open reading frame (ORF; Gaspin et al., 2000; Dennis et al., 2001; Randau, 2012; Tripp et al., 2017; Lui et al., 2018). Twenty-five percent of genes show overlap with a 3' end and carry the stop codon of the upstream gene within their sequence. Though the stop codon can be found in any of the four conserved motifs, in almost 50% of cases it was located in the C box motif (Tripp et al., 2017). Notably, the stop codon “UGA” is found in most of the C box and D box motifs and only a few nucleotide changes separate it from evolving into a k-turn element. Therefore, it was proposed that the start or stop codons of overlapping genes are responsible for the accelerated evolution of k-turn motifs in C/D box sRNA genes (Tripp et al., 2017). A 5' overlap was identified for 7% of the investigated C/D box sRNA genes. Similarly, the start codon of an overlapping downstream coding region was found to be located within different parts of C/D box sRNA sequences, most commonly however, in the guide sequence or downstream of the D box motif (Tripp et al., 2017). Analyses of the impact of these overlaps on the transcription rate of a reporter gene revealed neutral or only slightly negative effects for 3' overlapping C/D box sRNAs genes. However, a 5' overlap caused a significant reduction in transcription of the downstream gene, which is in agreement with the rare presence of this gene arrangement in nature (Tripp et al., 2017). In some cases, this scenario might result in the formation of pseudogene sequences downstream of C/D box sRNA genes.

A significant fraction of C/D box sRNA genes was found to occur in clusters of two or three genes indicating polycistronic transcription. Several dicistronic transcripts, including examples of tRNA- C/D box sRNA fusions, were identified (Tripp et al., 2017). In these cases, different C/D box sRNAs were, for example, found to be located directly downstream of genes coding for tRNASer in *I. hospitalis*, tRNAPro in *T. tenax*, and tRNAVal in *N. equitans*. Consequently, tRNA 3' maturation is suggested to generate the 5' terminus of the respective C/D box sRNAs. In some cases, C/D box sRNAs were also found to be located within tRNA introns and shown to mediate tRNA methylation *in cis* (D’Orval et al., 2001). The majority of the remaining genes are located in intergenic regions and some of them exhibit the aforementioned, conserved motifs, indicating the presence of an independent promoter. Others are located up- or downstream of neighboring protein-coding genes at a distance of less than 25 nt. Consequently, most C/D box sRNA genes do not require independent promoters, as they are part of longer precursor transcripts that are subsequently processed into mature C/D box sRNAs (Tripp et al., 2017).

Mutational analyses of *S. acidocaldarius* upstream and downstream regions of a C/D box sRNA gene revealed that these surrounding sequences can be changed without affecting C/D box sRNA maturation. Instead, the presence of the conserved, internal box motifs responsible for forming the k-turn and k-loop structures was found to be essential (Tripp et al., 2017). These observations suggest that the insertion of a C/D box sRNA gene into a transcriptionally active genome context is sufficient to obtain mature C/D box sRNAs. In this model, C/D box sRNP formation would result in the protection of the C/D box sRNA body *via* protein-RNA contacts while the exposed RNA termini would gradually be processed by cellular nucleases and/or chemical RNA degradation at elevated temperatures. In addition, interactions with RNA ligases would then yield fractions of circularized C/D box sRNAs without accessible RNA termini.

### Identification of Guide Sequences

The conserved box C and box D sequences of C/D box RNAs and their evenly spaced arrangement into two k-turns for L7Ae binding allow for the computational prediction of C/D box sRNA genes among archaeal sequences. One of the first programs that used these features to scan genomes for snoRNAs and their putative methylation targets in rRNA is snoScan (Lowe and Eddy, 1999). This program applies probabilistic models of snoRNAs and initially identified 22 novel guides in *Saccharomyces cerevisiae*. Although the initial development of snoScan focused on the prediction of rRNA methylation sites, different RNA sequences can also be used for target prediction. Another tool that utilizes probabilistic models is snoSeeker (Yang et al., 2006). This program searches for box C and box D elements, terminal stem pairing and, optionally, target sequences, enabling prediction of both guide and orphan sRNAs. The algorithm SnoReport utilizes support vector machines (SVM) and RNA secondary structure prediction to identify C/D box sRNA sequences (Hertel et al., 2008; de Araujo Oliveira et al., 2016). A more recent version, SnoReport 2.0, takes advantage of features of known C/D box sRNAs detected in invertebrates to improve its SVM during the training phase. In addition, a k-turn test, in which the predicted sRNAs must present G.A dinucleotides in box C and D, at least one uridine for the U-U pair and a Watson-Crick base pair between the sixth nt of the C box and the first nt of the D box significantly reduced the number of false positives (de Araujo Oliveira et al., 2016). Additionally, snoStrip (Bartschat et al., 2014) is a comprehensive pipeline that applies the following steps: first, a sequence-based homology search is performed using BLASTn (Altschul et al., 1990) and further complemented with the generation of covariance models. Next, the detection of characteristic C and D box motifs is performed through temporary alignments using MUSCLE (Edgar, 2004). If the location of a box motif agrees in all alignments, the position is annotated as a candidate box sequence. After defining conserved sequence elements, a secondary structure analysis is employed to ensure that only correctly folded C/D box RNAs are further analyzed. Finally, prediction of the putative targets is achieved using Plexy, a tool that calculates the optimal thermodynamic interactions of a C/D box sRNA with candidate targets (Kehr et al., 2011). As the repertoire of sequenced C/D box sRNAs increases, several databases have been created to categorize these molecules, including the Plant snoRNA database (Brown et al., 2001). For archaea, two databases can be highlighted: Rfam and snoRNAdb (Lowe and Eddy, 1999; Omer et al., 2000; Kalvari et al., 2021). The Rfam database uses a generalized search based on covariance models to annotate a wide diversity of non-coding RNAs, including C/D box sRNAs, that are conserved in three or more species. The database snoRNAdb compiles homologs of C/D box sRNAs that were predicted for crenarchaeal and euryarchaeal species, while also providing information about their putative targets.

Even though these tools and different strategies are available for C/D box sRNA prediction, this class of RNA is still underrepresented in archaeal annotations (Gardner et al., 2010) and only a combination of RNA-seq analyses, comparative genomics and computational methods allow for complete C/D box sRNA identification (Lui et al., 2018). Since most prediction algorithms were developed using eukaryotic C/D box sRNAs as training sets, it is hypothesized that features which are exclusive to archaeal C/D box sRNAs are absent, therefore impacting the overall efficiency and reliability of the predictions. Here, the lower degree of conservation of C' and D' boxes in eukaryotes in comparison to archaea represents one clear difference (Yang et al., 2020). The recent increase in the availability of archaeal transcriptome datasets is an asset to expand the repertoire of hand-curated C/D box sRNAs. Utilizing experimentally validated datasets, tools that are based on pre-generated covariance models (e.g. INFERNAL – cmsearch) can take advantage of the conserved C' and D' motifs to drastically increase the number of predicted C/D box sRNAs in Archaea (Lui et al., 2018). The reduction of the stringency of the search parameters for C/D box sRNA genes results in increasing amounts of false positive sequences resembling C/D box sRNA genes. These hits can be viewed as sequence space with increased probability of evolving novel C/D box sRNA elements and might impact the dynamics of guide sequence generation.

## Discussion

C/D box sRNAs were early found to possess other functions besides their established role in the 2'-*O*-methylation of rRNA and tRNA (Dennis et al., 2001). C/D box sRNAs of yeast and eukaryotes (especially humans) have been shown to be involved in diverse functions including rRNA processing, RNA base acetylation, regulation of mRNA 3' processing, and alternative pre-mRNA splicing (Kass et al., 1990; Falaleeva et al., 2016; Huang et al., 2017; Sharma et al., 2017b). Recently, it was shown that C/D box snoRNA-guided methylation of mRNA regulates protein expression and enzyme activity (Elliott et al., 2019). Additionally, it was revealed that many snoRNAs are processed into shorter forms such as miRNA (called sno-miRNA) and efficiently exert gene regulatory functions (Brameier et al., 2011). It was also recently discovered that snoRNAs retained in longer RNAs can interact with non-canonical proteins and act as a decoy, thereby hindering their activity (Bergeron et al., 2020). In fact, the influence of C/D box snoRNAs in the human metabolism is very significant: the C/D box snoRNA *U60* is involved in intracellular cholesterol trafficking and regulation of cholesterol homeostasis (Brandis et al., 2013). Lack of expression of the C/D box snoRNA cluster *SNORD116* causes Prader-Willi-Syndrome, a neurobehavioral disorder manifesting itself in hyperphagia and leading to morbid obesity (Ding et al., 2008; Duker et al., 2010). Furthermore, the snoRNA *U50* was found to be deleted in several common cancers, with a particularly strong association in breast cancer and prostate cancer (Dong et al., 2008, 2009; Siprashvili et al., 2015). With an evident link between snoRNAs and human cancer and other systemic diseases being established, a strong resurgence of eukaryotic snoRNA research has been noted. New findings in this area continually expand our knowledge of diverse snoRNA functions and have most recently been reviewed by Deogharia and Majumder (2019), Liang et al. (2019) and Bratkovič et al. (2020).

Using RiboMethSeq to analyze the 2'-*O*-methylation patterns on eukaryotic rRNAs, it was shown that a knockdown of the methyltransferase fibrillarin (FBL) in HeLa-cells leads to a site-specific decrease of methylation levels. Affected sites were identified in conserved and/or functionally important regions of the ribosome, like its “core,” close to the A- and P-sites, the intersubunit bridges and the peptide exit tunnel, while 2'-*O*-Me sites close to the peptidyl transferase center were not subject to variation in methylation levels upon FBL knockdown (Erales et al., 2017). Another study from the same year mapping 2'-*O*-methylation sites vulnerable to fibrillarin depletion on human rRNAs, also investigated the C/D box sRNAs whose guide sequences target these “vulnerable” methylation sites. However, these studies did not find a direct correlation between the sites with a variable methylation level and abundance of the sRNAs which target them (Sharma et al., 2017a). More recently, RiboMethSeq was adapted to map 2'-*O*-methylation sites on rRNAs in human breast cancer samples (Marcel et al., 2020). Here, the identified methylation sites were divided into two classes: one class encompassing a larger group of rRNA 2'-*O*-methylation sites with a low inter-patient variability, termed “stable” sites, and a second class encompassing a smaller group of rRNA 2'-*O*-methylation sites with a high inter-patient variability in methylation levels, termed “variable” sites. These stable sites were found to be located in the decoding center, the peptidyl transferase center and the polypeptide exit tunnel, while the variable sites were located in layers 1 or 2 nt away from these functional regions. Furthermore, it is suggested that the 2'-*O*-methylation levels at the variable rRNA sites are associated with breast cancer subtype and tumor grade, indicating that not only tumor size but also the pattern of rRNA 2'-*O*-methylation influences factors like tumor aggressiveness and patient survival (Marcel et al., 2020).

Additional functional roles of archaeal C/D box sRNAs are likely also to be discovered, which is supported by the existence of many “orphan” C/D box sRNA guides in archaea without easily detectable complementary methylation targets. Guide sequences of C/D box sRNAs define the methylation landscape of their hybridization targets. As these targets are mostly highly conserved rRNA molecules, it is initially surprising to see that C/D box sRNA do not exhibit a similar degree of conservation. As described in Ribosomal Targets section, ubiquitous methylation of functionally and structurally important rRNA regions can be achieved by different sets of C/D box sRNAs with varied guide sequences and guide sequence pairs. This dynamic evolution of guides has been analyzed in detail in six *Pyrobaculum* species containing 526 different C/D box sRNAs that were organized into 110 homologous families (Lui et al., 2018). At the genus level, less than two-thirds of the predicted targets were found to be conserved among the six *Pyrobaculum* species and guide sequences exhibited short insertions, deletions or substitutions. In the *Pyrobaculum* species dataset, 28% of guides showed no significant complementarity to potential RNA targets (Lui et al., 2018). As C/D box sRNA genes often overlap with adjacent genes that provide promoter elements and processing signals, it is also possible that the overlapping sequence results in the creation of an orphan guide sequence that is paired with a second guide that provides methylation benefits for the cell. Therefore, the presence of orphan guide sequences can partly be considered to be a consequence of the plasticity of the genomic context of C/D box sRNA genes. Here, it remains to be understood why C/D box sRNAs exhibit dynamic scenarios of polycistronic transcriptional units with different mRNA and tRNA partners. In mammalian cells, snoRNAs have been found in retroposable elements and it was proposed that retroposition followed by genetic drift would be able to increase snoRNA diversity and change their modification landscape (Weber, 2006). Mobile features of archaeal C/D box sRNA genes remain to be discovered.

## Author Contributions

RB, J-VG-F, and LR conceptualized and wrote the manuscript. All authors contributed to the article and approved the submitted version.

### Conflict of Interest

The authors declare that the research was conducted in the absence of any commercial or financial relationships that could be construed as a potential conflict of interest.

## References

[ref1] Aittaleb M. Rashid R. Chen Q. Palmer J. R. Daniels C. J. Li H. (2003). Structure and function of archaeal box C/D sRNP core proteins. Nat. Struct. Biol. 10, 256–263. 10.1038/nsb905, PMID: 12598892

[ref2] Altschul S. F. Gish W. Miller W. Myers E. W. Lipman D. J. (1990). Basic local alignment search tool. J. Mol. Biol. 215, 403–410. 10.1016/S0022-2836(05)80360-2, PMID: 2231712

[ref3] Auffinger P. Westhof E. (1997). Rules governing the orientation of the 2'-hydroxyl group in RNA. J. Mol. Biol. 274, 54–63. 10.1006/jmbi.1997.1370, PMID: 9398515

[ref4] Ban N. Nissen P. Hansen J. Moore P. B. Steitz T. A. (2000). The complete atomic structure of the large ribosomal subunit at 2.4 Å resolution. Science 289, 905–920. 10.1126/science.289.5481.905, PMID: 10937989

[ref5] Barbezier N. Canino G. Rodor J. Jobet E. Saez-Vasquez J. Marchfelder A.. (2009). Processing of a dicistronic tRNA-snoRNA precursor: combined analysis in vitro and in vivo reveals alternate pathways and coupling to assembly of snoRNP. Plant Physiol. 150, 1598–1610. 10.1104/pp.109.137968, PMID: 19420328 PMC2705039

[ref6] Bartschat S. Kehr S. Tafer H. Stadler P. F. Hertel J. (2014). SnoStrip: a snorna annotation pipeline. Bioinformatics 30, 115–116. 10.1093/bioinformatics/btt604, PMID: 24174566

[ref7] Bergeron D. Fafard-Couture É. Scott M. S. (2020). Small nucleolar RNAs: continuing identification of novel members and increasing diversity of their molecular mechanisms of action. Biochem. Soc. Trans. 48, 645–656. 10.1042/BST20191046, PMID: 32267490 PMC7200641

[ref8] Bleichert F. Baserga S. J. (2010). Dissecting the role of conserved box C/D sRNA sequences in di-sRNP assembly and function. Nucleic Acids Res. 38, 8295–8305. 10.1093/nar/gkq690, PMID: 20693534 PMC3001065

[ref9] Bleichert F. Gagnon K. T. Brown B. A. II Maxwell E. S. Leschziner A. E. Unger V. M.. (2009). A dimeric structure for archaeal box C/D small ribonucleoproteins. Science 325, 1384–1388. 10.1126/science.1176099, PMID: 19745151 PMC2975540

[ref10] Bortolin M. L. Bachellerie J. P. Clouet-d’Orval B. (2003). In vitro RNP assembly and methylation guide activity of an unusual box C/D RNA, cis-acting archaeal pre-tRNATrp. Nucleic Acids Res. 31, 6524–6535. 10.1093/nar/gkg860, PMID: 14602911 PMC275556

[ref11] Bower-Phipps K. R. Taylor D. W. Wang H. W. Baserga S. J. (2012). The box C/D sRNP dimeric architecture is conserved across domain archaea. RNA 18, 1527–1540. 10.1261/rna.033134.112, PMID: 22753779 PMC3404373

[ref12] Brameier M. Herwig A. Reinhardt R. Walter L. Gruber J. (2011). Human box C/D snoRNAs with miRNA like functions: expanding the range of regulatory RNAs. Nucleic Acids Res. 39, 675–686. 10.1093/nar/gkq776, PMID: 20846955 PMC3025573

[ref13] Brandis K. A. Gale S. Jinn S. Langmade S. J. Dudley-Rucker N. Jiang H.. (2013). Box C/D small nucleolar RNA (snoRNA) U60 regulates intracellular cholesterol trafficking. J. Biol. Chem. 288, 35703–35713. 10.1074/jbc.M113.488577, PMID: 24174535 PMC3861622

[ref14] Bratkovič T. Bozič J. Rogelj B. (2020). Functional diversity of small nucleolar RNAs. Nucleic Acids Res. 48, 1627–1651. 10.1093/nar/gkz1140, PMID: 31828325 PMC7038934

[ref15] Brown J. W. S. Clark G. P. Leader D. J. Simpson C. G. Lowe T. (2001). Multiple snoRNA gene clusters from *Arabidopsis*. RNA 7, 1817–1832. PMID: 11780637 PMC1370220

[ref16] Caffarelli E. Arese M. Santoro B. Fragapane P. Bozzoni I. (1994). In vitro study of processing of the intron-encoded U16 small nucleolar RNA in *Xenopus laevis*. Mol. Cell. Biol. 14, 2966–2974. 10.1128/mcb.14.5.2966, PMID: 7513048 PMC358664

[ref17] Caldas T. Binet E. Bouloc P. Richarme G. (2000). Translational defects of *Escherichia coli* mutants deficient in the Um2552 23S ribosomal RNA methyltransferase RrmJ/FTSJ. Biochem. Biophys. Res. Commun. 271, 714–718. 10.1006/bbrc.2000.2702, PMID: 10814528

[ref18] Cate J. H. Yusupov M. M. Yusupova G. Z. Earnest T. N. Noller H. F. (1999). X-ray crystal structures of 70S ribosome functional complexes. Science 285, 2095–2104. 10.1126/science.285.5436.2095, PMID: 10497122

[ref19] D’Orval B. C. Bortolin M. L. Gaspin C. Bachellerie J. P. (2001). Box C/D RNA guides for the ribose methylation of archaeal tRNAs. The tRNATrp intron guides the formation of two ribose-methylated nucleosides in the mature tRNATrp. Nucleic Acids Res. 29, 4518–4529. 10.1093/nar/29.22.4518, PMID: 11713301 PMC92551

[ref20] Danan M. Schwartz S. Edelheit S. Sorek R. (2012). Transcriptome-wide discovery of circular RNAs in Archaea. Nucleic Acids Res. 40, 3131–3142. 10.1093/nar/gkr1009, PMID: 22140119 PMC3326292

[ref21] de Araujo Oliveira J. V. Costa F. Backofen R. Stadler P. F. Machado Telles Walter M. E. Hertel J. (2016). SnoReport 2.0: new features and a refined support vector machine to improve snoRNA identification. BMC Bioinformatics 17:464. 10.1186/s12859-016-1345-6, PMID: 28105919 PMC5249026

[ref22] Decatur W. A. Fournier M. J. (2002). rRNA modifications and ribosome function. Trends Biochem. Sci. 27, 344–351. 10.1016/S0968-0004(02)02109-6 12114023

[ref23] Dennis P. P. Omer A. Lowe T. (2001). A guided tour: small RNA function in *Aarchaea*. Mol. Microbiol. 40, 509–519. 10.1046/j.1365-2958.2001.02381.x, PMID: 11359559

[ref24] Dennis P. P. Tripp V. Lui L. Lowe T. Randau L. (2015). C/D box sRNA-guided 2’-*O*-methylation patterns of archaeal rRNA molecules. BMC Genomics 16:632. 10.1186/s12864-015-1839-z, PMID: 26296872 PMC4644070

[ref25] Deogharia M. Majumder M. (2019). Guide snoRNAs: Drivers or passengers in human disease? Biology 8, 1–16. 10.3390/biology8010001, PMID: 30577491 PMC6466398

[ref26] Deryusheva S. Gall J. G. (2019). Small, smaller, smallest: minimal structural requirements for a fully functional box C/D modification guide RNA. Biomolecules 9:457. 10.3390/biom9090457, PMID: 31500270 PMC6770171

[ref27] Dieci G. Preti M. Montanini B. (2009). Eukaryotic snoRNAs: a paradigm for gene expression flexibility. Genomics 94, 83–88. 10.1016/j.ygeno.2009.05.002, PMID: 19446021

[ref28] Ding F. Li H. H. Zhang S. Solomon N. M. Camper S. A. Cohen P.. (2008). SnoRNA Snord116 (Pwcr1/MBll-85) deletion causes growth deficiency and hyperphagia in mice. PLoS One 3:e1709. 10.1371/journal.pone.0001709, PMID: 18320030 PMC2248623

[ref29] Dong X. Y. Guo P. Boyd J. Sun X. Li Q. Zhou W.. (2009). Implication of snoRNA U50 in human breast cancer. J. Genet. Genomics 36, 447–454. 10.1016/S1673-8527(08)60134-4 19683667 PMC2854654

[ref30] Dong X. Y. Rodriguez C. Guo P. Sun X. Talbot J. T. Zhou W.. (2008). SnoRNA U50 is a candidate tumor-suppressor gene at 6q14.3 with a mutation associated with clinically significant prostate cancer. Hum. Mol. Genet. 17, 1031–1042. 10.1093/hmg/ddm375 18202102 PMC2923223

[ref31] Duker A. L. Ballif B. C. Bawle E. V. Person R. E. Mahadevan S. Alliman S.. (2010). Paternally inherited microdeletion at 15q11.2 confirms a significant role for the SNORD116 C/D box snoRNA cluster in Prader-Willi syndrome. Eur. J. Hum. Genet. 18, 1196–1201. 10.1038/ejhg.2010.102, PMID: 20588305 PMC2987474

[ref32] Edgar R. C. (2004). MUSCLE: multiple sequence alignment with high accuracy and high throughput. Nucleic Acids Res. 32, 1792–1797. 10.1093/nar/gkh340, PMID: 15034147 PMC390337

[ref33] Elliott B. A. Ho H. T. Ranganathan S. V. Vangaveti S. Ilkayeva O. Abou Assi H.. (2019). Modification of messenger RNA by 2'-*O*-methylation regulates gene expression in vivo. Nat. Commun. 10:3401. 10.1038/s41467-019-11375-7, PMID: 31363086 PMC6667457

[ref34] Englert M. Sheppard K. Aslanian A. Yates J. R. Söll D. (2011). Archaeal 3'-phosphate RNA splicing ligase characterization identifies the missing component in tRNA maturation. Proc. Natl. Acad. Sci. U. S. A. 108, 1290–1295. 10.1073/pnas.1018307108, PMID: 21209330 PMC3029724

[ref35] Erales J. Marchand V. Panthu B. Gillot S. Belin S. Ghayad S. E.. (2017). Evidence for rRNA 2'-*O*-methylation plasticity: control of intrinsic translational capabilities of human ribosomes. Proc. Natl. Acad. Sci. U. S. A. 114, 12934–12939. 10.1073/pnas.1707674114, PMID: 29158377 PMC5724255

[ref36] Falaleeva M. Pages A. Matuszek Z. Hidmi S. Agranat-Tamir L. Korotkov K.. (2016). Dual function of C/D box small nucleolar RNAs in rRNA modification and alternative pre-mRNA splicing. Proc. Natl. Acad. Sci. U. S. A. 113, E1625–E1634. 10.1073/pnas.1519292113, PMID: 26957605 PMC4812717

[ref37] Falaleeva M. Welden J. R. Duncan M. J. Stamm S. (2017). C/D-box snoRNAs form methylating and non-methylating ribonucleoprotein complexes: old dogs show new tricks. BioEssays 39:201600264. 10.1002/bies.201600264, PMID: 28505386 PMC5586538

[ref38] Filipowicz W. Kiss T. (1993). Structure and function of nucleolar snRNPs. Mol. Biol. Rep. 18, 149–156. 10.1007/BF00986770, PMID: 7694081

[ref39] Gagnon K. T. Zhang X. Qu G. Biswas S. Suryadi J. Brown B. A.. (2010). Signature amino acids enable the archaeal L7Ae box C/D RNP core protein to recognize and bind the K-loop RNA motif. RNA 16, 79–90. 10.1261/rna.1692310, PMID: 19926724 PMC2802039

[ref40] Gardner P. P. Bateman A. Poole A. M. (2010). SnoPatrol: how many snoRNA genes are there? J. Biol. 9:4. 10.1186/jbiol211, PMID: 20122292 PMC2871523

[ref41] Gaspin C. Cavaillé J. Erauso G. Bachellerie J. -P. (2000). Archaeal homologs of eukaryotic methylation guide small nucleolar RNAs: lessons from the *Pyrococcus* genomes. J. Mol. Biol. 297, 895–906. 10.1006/JMBI.2000.3593, PMID: 10736225

[ref42] Graziadei A. Gabel F. Kirkpatrick J. Carlomagno T. (2020). The guide sRNA sequence determines the activity level of BOX C/D RNPs. elife 9, 1–27. 10.7554/eLife.50027, PMID: 32202498 PMC7089733

[ref43] Hansen M. A. Kirpekar F. Ritterbusch W. Vester B. (2002). Posttranscriptional modifications in the A-loop of 23S rRNAs from selected archaea and eubacteria. RNA 8, 202–213. 10.1017/S1355838202013365, PMID: 11911366 PMC1370243

[ref44] Helm M. (2006). Post-transcriptional nucleotide modification and alternative folding of RNA. Nucleic Acids Res. 34, 721–733. 10.1093/nar/gkj471, PMID: 16452298 PMC1360285

[ref45] Herschlag D. Eckstein F. Cech T. R. (1993). The importance of being ribose at the cleavage site in the Tetrahymena ribozyme reaction. Biochemistry 32, 8312–8321. 10.1021/bi00083a035, PMID: 7688573

[ref46] Hertel J. Hofacker I. I. Stadler P. F. (2008). SnoReport: computational identification of snoRNAs with unknown targets. Bioinformatics 24, 158–164. 10.1093/bioinformatics/btm464, PMID: 17895272

[ref47] Huang C. Shi J. Guo Y. Huang W. Huang S. Ming S.. (2017). A snoRNA modulates mRNA 3' end processing and regulates the expression of a subset of mRNAs. Nucleic Acids Res. 45, 8647–8660. 10.1093/nar/gkx651, PMID: 28911119 PMC5587809

[ref48] Hüttenhofer A. Kiefmann M. Meier-Ewert S. O’Brien J. Lehrach H. Bachellerie J. P.. (2001). Rnomics: an experimental approach that identifies 201 candidates for novel, small, non-messenger RNAs in mouse. EMBO J. 20, 2943–2953. 10.1093/emboj/20.11.2943, PMID: 11387227 PMC125495

[ref49] Jüttner M. Weiß M. Ostheimer N. Reglin C. Kern M. Knüppel R.. (2020). A versatile cis-acting element reporter system to study the function, maturation and stability of ribosomal RNA mutants in archaea. Nucleic Acids Res. 48, 2073–2090. 10.1093/nar/gkz1156, PMID: 31828323 PMC7038931

[ref50] Kalvari I. Nawrocki E. P. Ontiveros-Palacios N. Argasinska J. Lamkiewicz K. Marz M.. (2021). Rfam 14: expanded coverage of metagenomic, viral and microRNA families. Nucleic Acids Res. 49, D192–D200. 10.1093/nar/gkaa1047 33211869 PMC7779021

[ref51] Kass S. Tyc K. Steitz J. A. Sollner-Webb B. (1990). The U3 small nucleolar ribonucleoprotein functions in the first step of preribosomal RNA processing. Cell 60, 897–908. 10.1016/0092-8674(90)90338-F 2156625

[ref52] Kawai G. Yamamoto Y. Watanabe T. Yokoyama S. Kamimura T. Masegi T.. (1992). Conformational rigidity of specific pyrimidine residues in tRNA arises from posttranscriptional modifications that enhance steric interaction between the base and the 2'-hydroxyl group. Biochemistry 31, 1040–1046. 10.1021/bi00119a012, PMID: 1310418

[ref53] Kehr S. Bartschat S. Stadler P. F. Tafer H. (2011). PLEXY: efficient target prediction for box C/D snoRNAs. Bioinformatics 27, 279–280. 10.1093/bioinformatics/btq642, PMID: 21076148

[ref54] Kiss T. Filipowicz W. (1995). Exonucleolytic processing of small nucleolar RNAs from pre-mRNA introns. Genes Dev. 9, 1411–1424. 10.1101/gad.9.11.1411, PMID: 7797080

[ref55] Kiss-László Z. Henry Y. Bachellerie J. -P. Caizergues-Ferrer M. Kiss T. (1996). Site-specific ribose methylation of preribosomal RNA: a novel function for small nucleolar RNAs. Cell 85, 1077–1088. 10.1016/S0092-8674(00)81308-2, PMID: 8674114

[ref56] Kiss-Laszlo Z. Henry Y. H. Kiss T. (1998). Sequence and structural elements of methylation guide snoRNAs essential for site-specific ribose methylation of pre-rRNA. EMBO J. 17, 797–807. 10.1093/emboj/17.3.797, PMID: 9451004 PMC1170428

[ref57] Klein D. J. Schmeing T. M. Moore P. B. Steitz T. A. (2001). The kink-turn: a new RNA secondary structure motif. EMBO J. 20, 4214–4221. 10.1093/emboj/20.15.4214, PMID: 11483524 PMC149158

[ref58] Kruszka K. Barneche F. Guyot R. Ailhas J. Meneau I. Schiffer S.. (2003). Plant dicistronic tRNA-snoRNA genes: a new mode of expression of the small nucleolar RNAs processed by RNase Z. EMBO J. 22, 621–632. 10.1093/emboj/cdg040, PMID: 12554662 PMC140725

[ref59] Kuhn J. F. Tran E. J. Maxwell E. S. (2002). Archaeal ribosomal protein L7 is a functional homolog of the eukaryotic 15.5kD/Snu13p snoRNP core protein. Nucleic Acids Res. 30, 931–941. 10.1093/nar/30.4.931, PMID: 11842104 PMC100351

[ref60] Lapinaite A. Simon B. Skjaerven L. Rakwalska-Bange M. Gabel F. Carlomagno T. (2013). The structure of the box C/D enzyme reveals regulation of RNA methylation. Nature 502, 519–523. 10.1038/nature12581, PMID: 24121435

[ref61] Leader D. J. Clark G. P. Watters J. Beven A. F. Shaw P. J. Brown J. W. S. (1997). Clusters of multiple different small nucleolar RNA genes in plants are expressed as and processed from polycistronic pre-snoRNAs. EMBO J. 16, 5742–5751. 10.1093/emboj/16.18.5742, PMID: 9312032 PMC1170205

[ref62] Leader D. J. Sanders J. F. Waugh R. Shaw P. Brown J. W. S. (1994). Molecular characterisation of plant U14 small nucleolar RNA genes: closely linked genes are transcribed as polycistronic U14 transcripts. Nucleic Acids Res. 22, 5196–5203. 10.1093/nar/22.24.5196, PMID: 7816606 PMC332060

[ref63] Li S. G. Zhou H. Luo Y. P. Zhang P. Qu L. H. (2005). Identification and functional analysis of 20 box H/ACA small nucleolar RNAs (snoRNAs) from *Schizosaccharomyces pombe*. J. Biol. Chem. 280, 16446–16455. 10.1074/jbc.M500326200, PMID: 15716270

[ref64] Liang X. -H. Liu Q. Fournier M. J. (2009). Loss of rRNA modifications in the decoding center of the ribosome impairs translation and strongly delays pre-rRNA processing. RNA 15, 1716–1728. 10.1261/rna.1724409, PMID: 19628622 PMC2743053

[ref65] Liang J. Wen J. Huang Z. Chen X. Zhang B. Chu L. (2019). Small Nucleolar RNAs: insight into their function in cancer. Front. Oncol. 9:587. 10.3389/fonc.2019.00587, PMID: 31338327 PMC6629867

[ref66] Lin J. Lai S. Jia R. Xu A. Zhang L. Lu J.. (2011). Structural basis for site-specific ribose methylation by box C/D RNA protein complexes. Nature 469, 559–564. 10.1038/nature09688, PMID: 21270896

[ref67] Lischwe M. A. Ochs R. L. Reddy R. Cook G. Yeoman L. C. Tang E. M.. (1985). Purification and partial characterization of a nucleolar scleroderma antigen (Mr = 34,000; pI, 8.5) rich in NG, NG-dimethylarginine. J. Biol. Chem. 260, 14304–14310. 10.1016/S0021-9258(17)38718-5, PMID: 2414294

[ref68] Lowe T. M. Eddy S. R. (1999). A computational screen for methylation guide snoRNAs in yeast. Science 283, 1168–1171. 10.1126/science.283.5405.1168, PMID: 10024243

[ref69] Lui L. Lowe T. (2013). Small nucleolar RNAs and RNAguided post-transcriptional modification. Essays Biochem. 54, 53–77. 10.1042/BSE0540053, PMID: 23829527

[ref70] Lui L. M. Uzilov A. V. Bernick D. L. Corredor A. Lowe T. M. Dennis P. P. (2018). Methylation guide RNA evolution in archaea: structure, function and genomic organization of 110 C/D box sRNA families across six *Pyrobaculum* species. Nucleic Acids Res. 46, 5678–5691. 10.1093/nar/gky284, PMID: 29771354 PMC6009581

[ref71] Maden B. E. H. Corbett M. E. Heeney P. A. Pugh K. Ajuh P. M. (1995). Classical and novel approaches to the detection and localization of the numerous modified nucleotides in eukaryotic ribosomal RNA. Biochimie 77, 22–29. 10.1016/0300-9084(96)88100-4 7599273

[ref72] Marcel V. Kielbassa J. Marchand V. Natchiar K. S. Paraqindes H. Nguyen Van Long F.. (2020). Ribosomal RNA 2'*O*-methylation as a novel layer of inter-tumour heterogeneity in breast cancer. NAR Cancer 2, 1–12. 10.1093/narcan/zcaa036 PMC8210124 34316693

[ref73] Maxwell E. S. Fournier M. J. (1995). The small nucleolar RNAs. Annu. Rev. Biochem. 35, 897–934. 10.1146/annurev.bi.64.070195.004341 7574504

[ref74] Motorin Y. Helm M. (2010). TRNA stabilization by modified nucleotides. Biochemistry 49, 4934–4944. 10.1021/bi100408z 20459084

[ref75] Nolivos S. Carpousis A. J. Clouet-d’Orval B. (2005). The K-loop, a general feature of the *Pyrococcus* C/D guide RNAs, is an RNA structural motif related to the K-turn. Nucleic Acids Res. 33, 6507–6514. 10.1093/nar/gki962 16293637 PMC1289080

[ref76] Noon K. R. Bruenger E. McCloskey J. A. (1998). Posttranscriptional modifications in 16S and 23S rRNAs of the archaeal hyperthermophile *Sulfolobus solfataricus*. J. Bacteriol. 180, 2883–2888. 10.1128/jb.180.11.2883-2888.1998 9603876 PMC107253

[ref77] Ochs R. L. Lischwe M. A. Spohn W. H. Busch H. (1985). Fibrillarin: a new protein of the nucleolus identified by autoimmune sera. Biol. Cell. 54, 123–133. 10.1111/j.1768-322X.1985.tb00387.x, PMID: 2933102

[ref78] Ogle J. M. Brodersen D. E. Clemons J. Tarry M. J. Carter A. P. Ramakrishnan V. (2001). Recognition of cognate transfer RNA by the 30S ribosomal subunit. Science 292, 897–902. 10.1126/science.1060612, PMID: 11340196

[ref79] Omer A. D. Lowe T. M. Russell A. C. Ebhardt H. Eddy S. R. Dennis P. P. (2000). Homologs of small nucleolar RNAs in Archaea. Science 288, 517–522. 10.1126/science.288.5465.517, PMID: 10775111

[ref80] Omer A. D. Ziesche S. Ebhardt H. A. Dennis P. P. (2002). In vitro reconstitution and activity of a C/D box methylation guide ribonucleoprotein complex. Proc. Natl. Acad. Sci. U. S. A. 99, 5289–5294. 10.1073/pnas.082101999, PMID: 11959980 PMC122762

[ref81] Pelczar P. Filipowicz W. (1998). The host gene for Intronic U17 small Nucleolar RNAs in mammals has no protein-coding potential and is a member of the 5'-terminal oligopyrimidine gene family. Mol. Cell. Biol. 18, 4509–4518. 10.1128/mcb.18.8.4509 9671460 PMC109036

[ref82] Petrov A. S. Bernier C. R. Gulen B. Waterbury C. C. Hershkovits E. Hsiao C.. (2014). Secondary structures of rRNAs from all three domains of life. PLoS One 9:e88222. 10.1371/journal.pone.0088222, PMID: 24505437 PMC3914948

[ref83] Petrov A. S. Bernier C. R. Hershkovits E. Xue Y. Waterbury C. C. Hsiao C.. (2013). Secondary structure and domain architecture of the 23S and 5S rRNAs. Nucleic Acids Res. 41, 7522–7535. 10.1093/nar/gkt513, PMID: 23771137 PMC3753638

[ref84] Plagens A. Daume M. Wiegel J. Randau L. (2015). Circularization restores signal recognition particle RNA functionality in *Thermoproteus*. elife 4:e11623. 10.7554/eLife.11623.001, PMID: 26499493 PMC4731332

[ref85] Popow J. Englert M. Weitzer S. Schleiffer A. Mierzwa B. Mechtler K.. (2011). HSPC117 is the essential subunit of a human tRNA splicing ligase complex. Science 331, 760–764. 10.1126/science.1197847 21311021

[ref86] Randau L. (2012). RNA processing in the minimal organism *Nanoarchaeum equitans*. Genome Biol. 13:R63. 10.1186/gb-2012-13-7-r63 22809431 PMC3491384

[ref87] Sas-Chen A. Thomas J. M. Matzov D. Taoka M. Nance K. D. Nir R.. (2020). Dynamic RNA acetylation revealed by quantitative cross-evolutionary mapping. Nature 583, 638–643. 10.1038/s41586-020-2418-2 32555463 PMC8130014

[ref88] Schoemaker R. J. W. Gultyaev A. P. (2006). Computer simulation of chaperone effects of Archaeal C/D box sRNA binding on rRNA folding. Nucleic Acids Res. 34, 2015–2026. 10.1093/nar/gkl154. 16614451 PMC1435978

[ref89] Sharma S. Marchand V. Motorin Y. Lafontaine D. L. J. (2017a). Identification of sites of 2'-*O*-methylation vulnerability in human ribosomal RNAs by systematic mapping. Sci. Rep. 7, 1–15. 10.1038/s41598-017-09734-9. 28904332 PMC5597630

[ref90] Sharma S. Yang J. van Nues R. Watzinger P. Kötter P. Lafontaine D. L. J.. (2017b). Specialized box C/D snoRNPs act as antisense guides to target RNA base acetylation. PLoS Genet. 13:e1006804. 10.1371/journal.pgen.1006804, PMID: 28542199 PMC5464676

[ref91] Singh S. K. Gurha P. Tran E. J. Maxwell E. S. Gupta R. (2004). Sequential 2'-*O*-methylation of archaeal pre-tRNA Trp nucleotides is guided by the intron-encoded but trans-acting box C/D ribonucleoprotein of pre-tRNA. J. Biol. Chem. 279, 47661–47671. 10.1074/jbc.M408868200, PMID: 15347671

[ref92] Siprashvili Z. Webster D. E. Johnston D. Shenoy R. M. Ungewickell A. J. Bhaduri A.. (2015). The noncoding RNAs SNORD50A and SNORD50B bind K-Ras and are recurrently deleted in human cancer. Nat. Genet. 48, 53–58. 10.1038/ng.3452, PMID: 26595770 PMC5324971

[ref93] Starostina N. G. Marshburn S. Johnson L. S. Eddy S. R. Terns R. M. Terns M. P. (2004). Circular box C/D RNAs in *Pyrococcus furiosus*. Proc. Natl. Acad. Sci. U. S. A. 101, 14097–14101. 10.1073/pnas.0403520101 15375211 PMC521125

[ref94] Steitz J. A. Tycowski K. T. (1995). Small RNA chaperones for ribosome biogenesis. Science 270, 1626–1627. 10.1016/s1937-6448(10)84002-x 7502072

[ref95] Su A. A. H. Tripp V. Randau L. (2013). RNA-Seq analyses reveal the order of tRNA processing events and the maturation of C/D box and CRISPR RNAs in the hyperthermophile *Methanopyrus kandleri*. Nucleic Acids Res. 41, 6250–6258. 10.1093/nar/gkt317 23620296 PMC3695527

[ref96] Tomkuviene M. Ličyte J. Olendraite I. Liutkevičiute Z. Clouet-D’Orval B. Klimašauskas S. (2017). Archaeal fibrillarin-Nop5 heterodimer 2'-*O*-methylates RNA independently of the C/D guide RNP particle. RNA 23, 1329–1337. 10.1261/rna.059832.116 28576826 PMC5558902

[ref97] Tran E. Zhang X. Lackey L. Maxwell E. S. (2005). Conserved spacing between the box C/D and C'/D' RNPs of the archaeal box C/D sRNP complex is required for efficient 2'-*O*-methylation of target RNAs. RNA 11, 285–293. 10.1261/rna.7223405. 15661846 PMC1370718

[ref98] Tripp V. Martin R. Orell A. Alkhnbashi O. S. Backofen R. Randau L. (2017). Plasticity of archaeal C/D box sRNA biogenesis. Mol. Microbiol. 103, 151–164. 10.1111/mmi.13549. 27743417

[ref99] Trotta C. R. Miao F. Arn E. A. Stevens S. W. Ho C. K. Rauhut R.. (1997). The yeast tRNA splicing endonuclease: a tetrameric enzyme with two active site subunits homologous to the archaeal tRNA endonucleases. Cell 89, 849–858. 10.1016/S0092-8674(00)80270-6. 9200603

[ref100] Tycowski K. T. Aab A. Steitz J. A. (2004). Guide RNAs with 5† caps and novel box C/D snoRNA-like domains for modification of snRNAs in Metazoa. Curr. Biol. 14, 1985–1995. 10.1016/j.cub.2004.11.003 15556860

[ref101] Tycowski K. T. You Z. H. Graham P. J. Steitz J. A. (1998). Modification of U6 spliceosomal RNA is guided by other small RNAs. Mol. Cell 2, 629–638. 10.1016/S1097-2765(00)80161-6. 9844635

[ref102] Villa T. Ceradini F. Presutti C. Bozzoni I. (1998). Processing of the intron-encoded U18 small Nucleolar RNA in the yeast *Saccharomyces cerevisiae* relies on both exo- and endonucleolytic activities. Mol. Cell. Biol. 18, 3376–3383. 10.1128/mcb.18.6.3376 9584178 PMC108919

[ref103] Vitali P. Kiss T. (2019). Cooperative 2'-*o*-methylation of the wobble cytidine of human elongator tRNAmet(cat) by a nucleolar and a cajal bodyspecific box C/D RNP. Genes Dev. 33, 741–746. 10.1101/gad.326363.119. 31171702 PMC6601510

[ref104] Wang W. Kim R. Jancarik J. Yokota H. Kim S. H. (2001). Crystal structure of phosphoserine phosphatase from *Methanococcus jannaschii*, a hyperthermophile, at 1.8 Å resolution. Structure 9, 65–71. 10.1016/S0969-2126(00)00558-X 11342136

[ref105] Watkins N. J. Ségault V. Charpentier B. Nottrott S. Fabrizio P. Bachi A.. (2000). A common Core RNP structure shared between the small Nucleoar box C/D RNPs and the Spliceosomal U4 snRNP. Cell 103, 457–466. 11081632 10.1016/s0092-8674(00)00137-9

[ref106] Weber M. J. (2006). Mammalian small nucleolar RNAs are mobile genetic elements. PLoS Genet. 2:e205. 10.1371/journal.pgen.0020205, PMID: 17154719 PMC1687206

[ref107] Williams D. J. Boots J. L. Hall K. B. (2001). Thermodynamics of 2'-ribose substitutions in UUCG tetraloops. RNA 7, 44–53. 10.1017/S1355838201001558, PMID: 11214179 PMC1370067

[ref108] Wimberly B. T. Brodersen D. E. Clemons W. M. Morgan-Warren R. J. Carter A. P. Vonrheln C.. (2000). Structure of the 30S ribosomal subunit. Nature 407, 327–339. 10.1038/35030006. 11014182

[ref109] Wolff P. Villette C. Zumsteg J. Heintz D. Antoine L. Chane-Woon-Ming B.. (2020). Comparative patterns of modified nucleotides in individual tRNA species from a mesophilic and two thermophilic archaea. RNA 26, 1957–1975. 10.1261/RNA.077537.120 32994183 PMC7668247

[ref110] Xue S. Wang R. Yang F. Terns R. M. Terns M. P. Zhang X.. (2010). Structural basis for substrate placement by an archaeal box C/D ribonucleoprotein particle. Mol. Cell 39, 939–949. 10.1016/j.molcel.2010.08.022. 20864039 PMC3572848

[ref111] Yang Z. Wang J. Huang L. Lilley D. M. J. Ye K. (2020). Functional organization of box C/D RNA-guided RNA methyltransferase. Nucleic Acids Res. 48, 5094–5105. 10.1093/nar/gkaa247, PMID: 32297938 PMC7229835

[ref112] Yang J. H. Zhang X. C. Huang Z. P. Zhou H. Huang M. B. Zhang S.. (2006). snoSeeker: an advanced computational package for screening of guide and orphan snoRNA genes in the human genome. Nucleic Acids Res. 34, 5112–5123. 10.1093/nar/gkl672. 16990247 PMC1636440

[ref113] Yu G. Zhao Y. Li H. (2018). The multistructural forms of box C/D ribonucleoprotein particles. RNA 24, 1625–1633. 10.1261/rna.068312.118. 30254138 PMC6239191

